# Evolution and heterogeneity of multiple serotypes of Dengue virus in Pakistan, 2006–2011

**DOI:** 10.1186/1743-422X-10-275

**Published:** 2013-09-04

**Authors:** Carmen Koo, Amna Nasir, Hapuarachchige Chanditha Hapuarachchi, Kim-Sung Lee, Zahra Hasan, Lee-Ching Ng, Erum Khan

**Affiliations:** 1 Environmental Health Institute, National Environment Agency, [as part of its work as a] WHO Collaborating Centre for Reference and Research of Arbovirus and their Associated Vectors, 11, Biopolis Way, #06-05-08, 138667 Singapore, Singapore; 2 Department of Pathology Microbiology, Aga Khan University, Stadium Road, P. O. Box 3500, Karachi 74800, Pakistan

**Keywords:** Dengue, Evolution, Diversity, Molecular epidemiology, Phylogenetics, Genome, Pakistan

## Abstract

**Background:**

Even though dengue has been recognized as one of the major public health threats in Pakistan, the understanding of its molecular epidemiology is still limited. The genotypic diversity of Dengue virus (DENV) serotypes involved in dengue outbreaks since 2005 in Pakistan is not well studied. Here, we investigated the origin, diversity, genetic relationships and geographic distribution of DENV to understand virus evolution during the recent expansion of dengue in Pakistan.

**Methods:**

The study included 200 sera obtained from dengue-suspected patients from 2006 to 2011. DENV infection was confirmed in 94 (47%) sera by a polymerase chain reaction assay. These included 36 (38.3%) DENV-2, 57 DENV-3 (60.6%) and 1 DENV-4 (1.1%) cases. Sequences of 13 whole genomes (6 DENV-2, 6 DENV-3 and 1 DENV-4) and 49 envelope genes (26 DENV-2, 22 DENV-3 and 1 DENV-4) were analysed to determine the origin, phylogeny, diversity and selection pressure during virus evolution.

**Results:**

DENV-2, DENV-3 and DENV-4 in Pakistan from 2006 to 2011 shared 98.5-99.6% nucleotide and 99.3-99.9% amino acid similarity with those circulated in the Indian subcontinent during the last decade. Nevertheless, Pakistan DENV-2 and DENV-3 strains formed distinct clades characterized by amino acid signatures of NS2A-I116T + NS5-K861R and NS3-K590R + NS5-S895L respectively. Each clade consisted of a heterogenous virus population that circulated in Southern (2006–2009) and Northern Pakistan (2011).

**Conclusions:**

DENV-2, DENV-3 and DENV-4 that circulated during 2006–2011 are likely to have first introduced via the southern route of Pakistan. Both DENV-2 and DENV-3 have undergone in-situ evolution to generate heterogenous populations, possibly driven by sustained local DENV transmission during 2006–2011 periods. While both DENV-2 and DENV-3 continued to circulate in Southern Pakistan until 2009, DENV-2 has spread in a Northern direction to establish in Punjab Province, which experienced a massive dengue outbreak in 2011.

## Background

Dengue is an acute febrile illness with a clinical spectrum of mild flu-like illness (dengue fever or DF) to fatal complications of severe dengue hemorrhagic fever (DHF) and dengue shock syndrome (DSS). The disease is caused by the Dengue virus (DENV) complex that consists of 4 genetically and immunogenically distinct serotypes. DENV is a single stranded positive sense RNA virus with a 11.8 kb genome flanked by 5’ and 3’ untranslated regions and a single coding region for three structural proteins: capsid (C), the precursor of membrane (prM) and envelope (E); and seven nonstructural proteins: NS1, NS2A, NS2B, NS3, NS4A, NS4B and NS5 [1].

DENV is transmitted to humans through the bites of infected female mosquitoes of *Aedes aegypti* and *Ae. albopictus.* The transmission of DENV has increased in recent years in urban and semi-urban endemic settings, especially in Americas, South and South-east Asia and the Western Pacific. The magnitude, distribution, and clinical severity of dengue outbreaks have been alarmingly high in countries such as India [2], Sri Lanka [3], Nepal [4] Bangladesh [5] and Pakistan [6] in the Indian subcontinent during the last decade.

Pakistan is endemic to all four serotypes of DENV circulating throughout the year with a peak incidence during the post monsoon period between October and December [7,8]. Factors such as crowded cities, unsafe drinking water, inadequate sanitation and large number of refugees facilitate the spread of dengue in different parts of the country, resulting in increased morbidity and mortality. It is believed that DENV was first introduced into Pakistan through the importation of tyres containing eggs of infected mosquitoes at Karachi sea port [9]. Although serological evidence of DENV infections in Pakistan dates back to 1968 [10], the first confirmed outbreak associated with DHF occurred in the southern Pakistan city of Karachi in 1994 [11]. Serological studies confirmed the circulation of both DENV-1 and DENV-2 during the 1994 outbreak [12]. Since then, the disease has emerged as a major public health problem in the country [13]. DENV-3 was first reported during the 2005–2006 outbreak in Karachi [14,15]. By 2007, dengue started to emerge in the Northern Pakistan. DENV-2 and DENV-3 have been the dominant serotypes in Lahore from 2007 to 2009 [16]. Dengue showed a resurgence in November 2010, especially in Sindh, Punjab and Khyber Pakhtunkhwa regions subsequent to massive floods in July same year [6]. The outbreak escalated in Lahore, Punjab, in 2011 with 22,562 cases and 363 deaths due to severe DHF [6].

So far, there have been a few, comprehensive molecular epidemiological studies that describe DENV circulating in Pakistan. There is only one genome-wide analysis that describes Pakistani DENV-2 circulated during the 2011 outbreak [17]. The studies of molecular epidemiology and evolutionary genetics are important to predict the origin and spread of viruses, to strengthen our understanding on the pathogenesis of disease, the cause of epidemics and the genetic basis of virulence. RNA viruses such as DENV evolve rapidly [18] and on rare occasions, certain mutations lead to phenotypic changes in the viruses that alter their potential to cause outbreaks associated with severe disease [19]. Genotypic characterization has been a useful tool in determining the evolutionary origin of the DENV, identifying the circulating virus strains in an endemic area and detecting the introduction of new genotypes. In recent years, envelope (E) gene sequencing has been widely used to assess the phylogenetic relationships among DENV isolates [20]. In Pakistan, sequencing of NS3 gene [21] and C-prM gene [14,16] junction has mainly been used for the genetic analyses of DENV. Khan and co-workers recently performed a more comprehensive analysis of DENV2 strains associated with 2011 outbreak in Lahore [17]. Nevertheless, the use of relatively short (up to 400 bp) and small number of sequences in available studies have limited the resolution of phylogenetic analyses aimed at determining the origin and evolution of DENV in Pakistan during the last decade.

Therefore, in the present study, we conducted a detailed molecular characterization of Pakistan DENV strains using the complete E gene and whole genome analyses. DENV isolates obtained from different parts of Pakistan between 2006 and 2011 were studied, in order to obtain a temporal evolutionary perspective of DENV in the country over a 6-year period. We describe the likely origin, evolution and geographic distribution of DENV that explain the recent changes of dengue epidemiology in Pakistan.

## Results and discussion

Out of 200 sera analysed, 94 (47%) were positive for DENV by polymerase chain reaction (PCR). These included 36 DENV-2 (38.3%), 57 DENV-3 (60.6%) and 1 DENV-4 (1.1%) cases (Table 1). None of the samples was positive for DENV-1. DENV could be isolated only from 25 PCR-positive sera (26.6%). These included 12 DENV-2 (48.0%), 12 DENV-3 (48.0%) and 1 DENV-4 (4.0%) cases. The reduced success rate of virus isolation in PCR positive sera is due to collection of the majority of sera during the late viraemic phase and further reduction in virus titres during long-term storage, transfer and freeze-thawing of samples. Among the DENV-2 samples, 7 were classified as DF, 16 as DHF and 3 as DSS, whereas for DENV-3, there were 18 DF and 4 DHF cases. Only 1 DENV-4 sample classified as DF was included in this study (Additional file 1).

**Table 1:** Number of dengue positive samples in study cohort during 2006-2011

**Collection year**	**No. of sera analysed**	**No. of positive samples**	**DENV Serotype**			
			**DENV-1**	**DENV-2**	**DENV-3**	**DENV-4**
2006	97	46	0	0	46	0
2007	3	3	0	0	3	0
2008	8	4	0	2	2	0
2009	61	28	0	21	6	1
2011	30	13	0	13	0	0
Unknown	1	0	0	0	0	0
**Total**	**200**	**94**	**0**	**36**	**57**	**1**

We detected only DENV-3 among sera collected during the 2006–2007 periods, whereas DENV-2, DENV-3 and DENV-4 were observed in sera collected after 2008. This is in agreement with previous findings that DENV-2 became dominant during the 2007–2009 periods [16]. The absence of DENV-1 in our cohort was not surprising as it has not been documented in Pakistan between 2006 and 2009 [9,16] and was a minor serotype in 2010 in Northern parts of the country [22]. During the 2006–2009 periods, 87.7% (n=71) of analysed sera were collected in Karachi, the capital of Sindh province in Southern Pakistan. On the other hand, 69.2% (n=9) of cases in 2011 were from Lahore in Punjab province of the Northern Pakistan where a major dengue outbreak was reported in the same year. This case distribution pattern reflects the dengue epidemiology in Pakistan, where the disease was initially restricted to the southern part of the country, particularly the port city of Karachi, and subsequently expanded to the north, causing major epidemics. The spread of dengue to other parts of the country is believed to be either due to the movement of people carrying the virus or mosquito eggs carrying vertically-transmitted DENV from dengue-affected areas [6].

In order to understand the origin and evolution of different DENV serotypes during their spread in Pakistan, we performed a detailed analysis of 13 full genomes (6 DENV-2, 6 DENV-3 and 1 DENV-4) and 49 envelope (E) gene sequences detected in Southern and Northern parts during the 2006–2011 period. All E gene sequences were obtained directly from PCR-positive sera and comprised 26 DENV-2, 22 DENV-3 and 1 DENV-4 strains. Full genome sequencing was performed on viruses isolated from sera.

### Characteristics of DENV-2 in Pakistan

**Figure 1:**
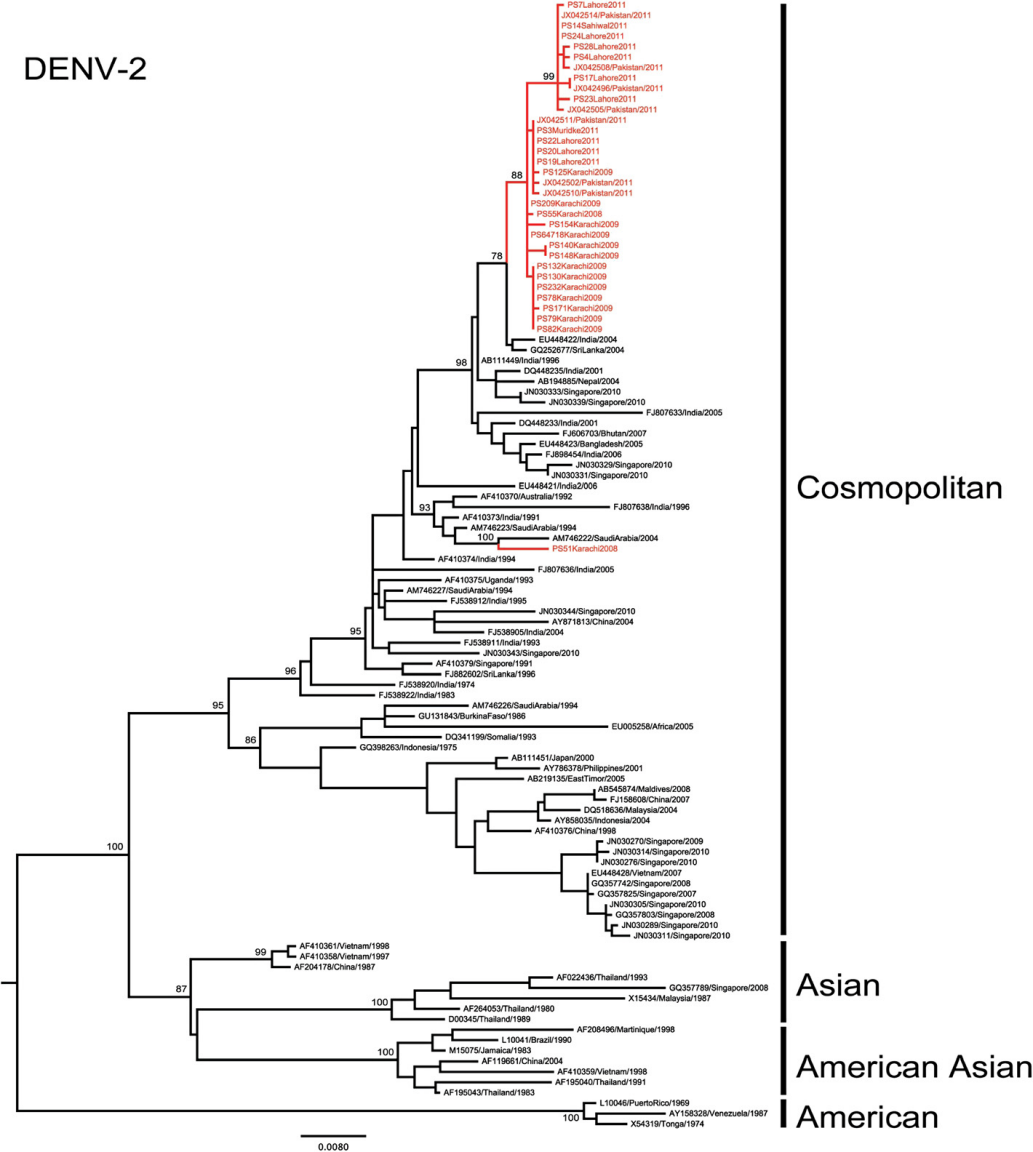
**Phylogenetic tree of DENV-2.** The maximum-likelihood tree was constructed based on the complete envelope gene sequences generated during this study and retrieved from GenBank. Sequences from Pakistan are highlighted in red. Figures on branches are bootstrap percentages. Only bootstrap values more than 75% are shown on the major nodes.

Phylogenetic analysis of 26 DENV-2 E gene sequences obtained between 2008 and 2011 revealed that all viruses belonged to Indian subcontinent lineage of the cosmopolitan genotype. Although there were more DHF compared to DF among the samples collected, there was no unique signature in the E gene sequences that could differentiate between DF and DHF/DSS samples. With the exception of 1 isolate (D2-PS51) that was closely related to a previous strain from Saudi Arabia, the remaining 25 isolates formed a distinct monophyletic clade with 78% bootstrap support (Figure 1). In DENV-2 whole genome analysis, the same clade achieved 100% bootstrap support, strengthening the credibility of the clade further (data not shown). This monophyletic clade included DENV-2 isolates obtained from 14 sera collected in Southern Pakistan (Karachi) in 2008 and 2009 as well as 11 sera collected in Northern Pakistan in 2011. Among complete E gene sequences available in GenBank database, these 25 viruses shared the highest nucleotide (98.8-99.5%) and amino acid (99.5-100%) similarity with those reported from Sri Lanka [GenBank: GQ252676 and GQ252677] and India [GenBank: DQ448231] during the 2003–04 periods. The nucleotide (99.1-99.4%) and amino acid (99.7-99.9%) similarity was even higher at whole genome level between Pakistan and 2003–2004 Indian/Sri Lankan strains. Furthermore, whole genome analysis revealed a unique combination of 8 amino acid substitutions that was shared between the monophyletic clade of Pakistan DENV-2 and Indian/Sri Lankan isolates (Table 2). These findings indicated the close genetic relationship among DENV-2 strains circulated in the Indian subcontinent during the last decade. Even though the earliest DENV-2 isolate of our study cohort was in 2008, estimation of time to the most recent common ancestor (TMRCA) dated the origin of Pakistan DENV-2 clade to early 2006 [95% highest probability density (HPD) 2004.5 – 2007.4]. Given the fact that genetically similar strains existed in the region even in 2003, it is likely that the 2008–2011 clade of Pakistan DENV-2 viruses descended from an ancestral strain that was previously circulating in other parts of the Indian subcontinent region. Further evolution in subsequent years has deviated Pakistan DENV-2 from its common ancestor to form a monophyletic clade by 2008. During this process, Pakistan DENV-2 strains have acquired 2 additional fixed amino acid substitutions (NS2A-I116T and NS5-K861R), of which NS5-K861R is unique to Pakistan strains (Table 2). In DENV-2, residue NS5-861 is a strictly conserved Lysine. NS5-861 resides in the “thumb” domain of the C-terminal end of RNA dependent RNA polymerase (RdRp) of DENV [23]. This residue sits outside structural elements of thumb domain that form the RNA template tunnel (residues 740–747 and 782–809) and a zinc binding site (residues 712, 714, 728 and 847). Therefore, even though the functional implications of the Lysine to Arginine substitution in our isolates are not clear, major structural changes are not expected as the K861R substitution does not alter the amino acid properties at the residue.

**Table 2:** Genome-wide mutation and selection pressure analysis of Pakistan DENV-2 isolates

**Polyprotein position**	**Gene**	**Gene position**	**Wild type**	**Post-2008 Pakistan isolates (n=5)**	**Other reports of similar substitutions**	**Mixed effect model of evolution (MEME) analysis**					
						**α**	**β**^**-**^	**q**^**-**^	**β**^**+**^	**P value**	**Log L**
130	prM	16	R	K	Sri Lanka2003-04^§^ & Pakistan [17]	1.87	0	0.97	91.05	0.15	−35.52
**184**	**prM**	**70**	**S**	**A**	**Pakistan**^**£**^[17] &**Malaysia (sylvatic)**[24,25]	**3.61**	**0**	**1**	**134.67**	**0.05**	**−41.97**
862	NS1	87	T	S	Sri Lanka2003-04^§^	1.25	0	0.97	6.49	0.74	−27.76
1160	NS2A	33	I	V	India2001-06^§^ & Sri Lanka 1996-2004^§^	0	1	0.06	0.33	0.66	−13.61
1243	NS2A	116	I	T	Thailand 1984 [26]	0	0	0.6	2.7	0.42	−40.58
2891	NS5	400	T	A	Sri Lanka 2003-04^§^	0	0	0.93	2.85	0.48	−10.8
2953	NS5	462	L	I	India2001-06^§^ & Sri Lanka 1996-04^§^	1.12	0	0.99	513.74	0.20	−38.62
3107	NS5	616	E	G	India2001-06^§^ & Sri Lanka 1996-2004^§^	0	1	0.62	0.85	0.44	−9.39
3122	NS5	631	S	G	India2001-06^§^ & Sri Lanka 1996-2004^§^	0.35	0.45	0.16	1.44	0.23	−32.15
**3352**	**NS5**	**861**	**K**	**R**	**Unique to Pakistan**^**£**^	**0**	**0**	**0.98**	**20.75**	**0.26**	**−11.06**
3367	NS5	876	N	S	India2001-06^§^ & Sri Lanka 1996-04^§^	0	0	0.98	19.81	0.19	−11.44

^§^Directly obtained from the GenBank database.

^£^Reported in this study.

Mutations under positive selection and those that are unique to Pakistan DENV-2 isolates are shown in bold.

Furthermore, our findings indicated an in-situ spatio-temporal evolutionary pattern of Pakistan DENV-2 during the period from 2008 to 2011. This was evident by the step-ladder pattern topology of the 2008–2011 Pakistan clade (Figure 1), in which the 2009 viruses in Southern Pakistan were ancestral to the distinct sub-clade (99% bootstrap support) formed by 2011 viruses in the North. This 2011 sub-clade possessed a unique substitution (prM-S70A) that was shown to be under the diversifying selection (p=0.05, Table 2) by the mixed effect model of evolution (MEME) method. In contrast, single likelihhod ancestor counting (SLAC), fixed effects likelihood (FEL) [27] and internal fixed effects likelihood (IFEL) [28] methods showed the same residue to be under purifying selection. MEME is one of the methods available in Hyphy package to perform the codon-based selection pressure analysis [29]. A simulation study using MEME has shown that MEME is preferable to other methods as it is capable of identifying not only pervasive positive selection at the level of an individual site, but also episodic (transient) positive selection affecting only a subset of lineages [30]. Episodic selection is often missed by other methods that rely on the mean non-synonymous to synonymous ratio (dN/dS) over a period of time [31]. Sequences from another recent study [17] have also shown prM-S70A to be fixed and unique to isolates from Lahore in 2011. Therefore, we postulate that amino acid residue prM-70 is likely to have been selected for during the DENV-2 outbreak in Northern Pakistan in 2011. As the support for diversifying selection in MEME analysis is marginally significant (p=0.05) and we obtained contrasting outputs from SLAC, FEL and IFEL methods, it is difficult to conclude whether the fixation of prM-S70A among Pakistani DENV-2 strains is either due to positive selection or genetic drift. Nevertheless, codon 70 constitutes the variable, middle amino acid residue of the N-glycosylation site sequon (N-S-T) of DENV-2 prM protein [1]. N-glycosylation of prM protein is believed to play an important role in virion assembly and transport before release [1]. The prM-70 residue is a strictly-conserved Serine in human DENV-2 isolates, except for two sylvatic viruses (N-A-T) reported from Malaysia in 2008 [24] and 1970 [25]. Therefore, whether this substitution from a hydrophilic Serine to a hydrophobic Alanine at prM-70 residue alters the conformation and thereby the functionality of the N-glycosylation site providing a selective advantage to DENV is of interest and necessitates further investigation.

As illustrated in Figure 1, virus populations from Southern and Northern areas are heterogenous, indicating the possibility of sustained virus transmission in both areas driving the viral diversity. While the majority of 2011 viruses belonged to a distinct clade, some remained within a 2009 virus cluster (Figure 1). Based on these observations, it is likely that DENV-2 first expanded in Southern Pakistan and subsequently spread to the Northern region where certain sub-population/s evolved further to become a distinct viral population in 2011. Therefore, the overall epidemiological understanding of South to North expansion of dengue in Pakistan is supported by our genetic analysis of DENV-2.

### Characteristics of DENV-3 in Pakistan

**Figure 2:**
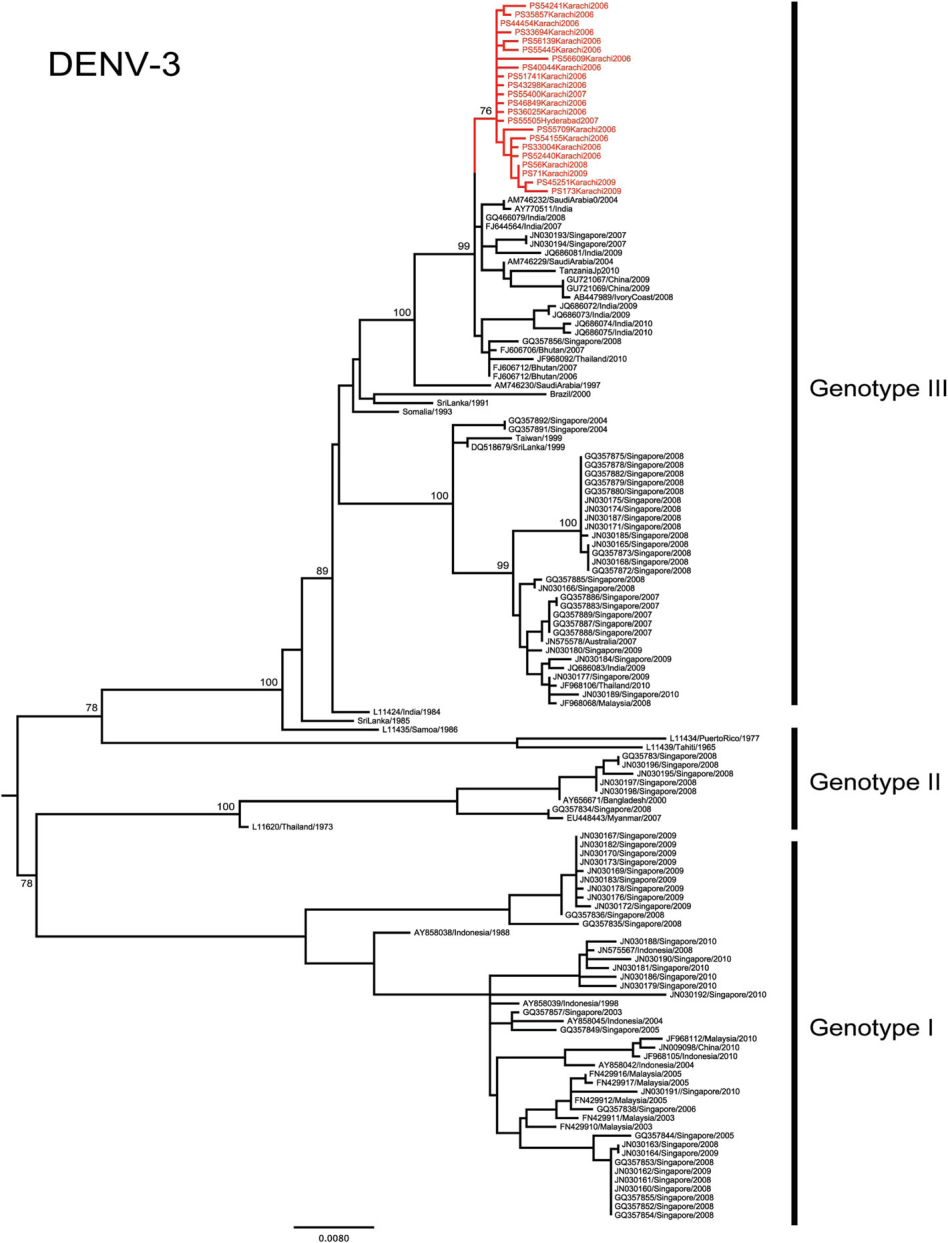
**Phylogenetic tree of DENV-3.** The maximum-likelihood tree was constructed based on the complete envelope gene sequences generated during this study and retrieved from GenBank. Sequences from Pakistan are highlighted in red. Figures on branches are bootstrap percentages. Only bootstrap values more than 75% are shown on the major nodes.

The emergence of DENV-3 in Pakistan was first reported during the 2005 DHF outbreak in Karachi [14]. Previous studies have revealed that DENV-3 sequences from the 2005 and 2006 outbreaks were most closely related to those circulating in India [14,32] and belonged to genotype III [16]. In support, all 22 DENV-3 E gene sequences from our study also belonged to the genotype III (Figure 2). Furthermore, all of our study isolates that were collected in southern Pakistan (Karachi and Hyderabad) from 2006 to 2009 shared the highest genetic similarity with two Indian DENV-3 strains [GenBank: AY770511 and FJ644564] and one Chinese strain [GenBank: GU363549]. Of these, one of the Indian strains [GenBank: AY770511] was reported in 2003 [32] whereas the remaining Indian [33] and Chinese strains were reported in 2007 and 2009 respectively. Therefore, it was evident that ancestral strains of Pakistan DENV-3 were present in the Indian subcontinent before their emergence in Pakistan during the 2005–2006 periods. At E gene level, nucleotide and amino acid similarities between the Pakistan and the Indian strain in 2003 [GenBank: AY770511] were 98.8-99.2% and 99.5-100%, respectively. The respective whole genome similarities between Pakistan DENV-3 isolates (n=6) and the same Indian strain were 98.9-99.2% (nucleotide) and 99.6-99.8% (amino acid). Sharing of a unique combination of 4 amino acid substitutions (NS1-L178M, NS4B-M151I, NS5-T188A and NS5-Q562L) between Pakistan and Indian isolates (Table 3) further supported the previous notion that Pakistan DENV-3 isolates are genetically related to those circulating in India in preceding years. Of these, NS1-L178M was found to be a positively selected site (p: <0.01. Table 3) in the MEME-based selection pressure analysis, suggesting a lineage-specific selective advantage of DENV-3 that circulated in the Indian subcontinent before 2009. SLAC, FEL and IFEL-based slection pressure analyses predicted the same site to be neutral. The residue 178 sits in domain II of flavivirus NS1 protein [34], adjacent to one of the 12 strictly conserved Cysteine residues (DENV-3-Cys179) that form disulfide linkages [1]. Disulfide bridges are believed to play an important role in NS1 dimerization [35]. Interestingly, residue 178 appears to follow a lineage-specific variability as DENV-3 genotype III lineage in South-east Asia (Vietnam, Cambodia, Thailand) shows a fixed NS1-L178S substitution. This observation, together with our data, indicates the likelihood of episodic diversifying selection at DENV-3 NS1-178 residue, as predicted by the MEME analysis for positive selection pressure.

**Table 3:** Genome-wide mutation and selection pressure analysis of Pakistan DENV-3 isolates

**Polyprotein position**	**Gene**	**Gene position**	**Wild type**	**Pakistan isolates (n=6)**	**Other reports of similar substitutions**	**Mixed effect model of evolution (MEME) analysis**					
						**α**	**β**^**-**^	**q**^**-**^	**β**^**+**^	**P value**	**Log L**
**951**	**NS1**	**178**	**L**	**M**	**India 2003–07**[32,33] &**China 2009**^**§**^	**1.83**	**0.36**	**0.99**	**1844.02**	**<0.01**	**−53.95**
**2063**	**NS3**	**590**	**K**	**R**	**Unique to Pakistan**^**£**^	**1.28**	**0**	**0.98**	**42.42**	**0.58**	**−17.20**
2393	NS4B	151	M	I	India 2003–07 [32,33] & China 2009^§^	1.35	0.16	1.00	0.31	1.00	−9.87
2678	NS5	188	T	A	India 2003–07 [32,33], China 2009^§^, Venezuela 2001-07^§^, Puerto Rico 2006^§^	0.85	0.38	0.99	707.57	0.21	−35.65
3052	NS5	562	Q	L	India 2003–07 [32,33], China 2009^§^, Colombia 2004^§^	1.74	1.00	0	2.65	0.64	−33.19
3385	NS5	895	S	L	Puerto-Rico 2002-03^§^, Colombia 2003^§^, Vietnam 2006^§^	2.00	0	0.99	35.8	0.47	−21.96

^§^Directly obtained from the GenBank database.

^£^Reported in this study.

Mutations under positive selection and those that are unique to Pakistan DENV-3 isolates are shown in bold.

Overall, these findings suggested an Indian subcontinent ancestry of 2006–2009 Pakistan DENV-3. Interestingly, E gene-based TMRCA analysis dated the origin of 2006–2009 Pakistan DENV-3 to late 2002 (95% HPD 2001.6-2004.0). The available evidence of a genetically closest Indian DENV-3 isolate in 2003, therefore, confirms the circulation of similar virus strains in other parts of the Indian subcontinent at the time of emergence of the common ancestor of the 2006–2009 DENV-3 strains in Pakistan. However, despite their high similarity with Indian strains, 2006–2009 Pakistan DENV-3 formed a distinct E gene cluster with 76% bootstrap support (Figure 2). In DENV-3 whole genome phylogeny, the bootstrap support of the same clade improved to 100% (data not shown). Our whole genome analysis revealed that this genetic deviation of Pakistan strains from Indian sub continent isolates was driven by two additional fixed amino acid substitutions (NS3-K590R and NS5-S895L). Of these, NS3-K590R is unique to Pakistan DENV-3 isolates. NS3-K590R falls within a loop structure between alpha helices 9 and 10 of flavivirus helicase domain 3 [36]. Even though domain 3 does not house major functional elements of helicase activity, it contributes to structural stability and the formation of RNA binding cleft [36-39]. Domain 3 is also believed to act as the RNA-dependent RNA polymerase binding site [36]. As the residue 590 does not carry any known structural significance, the effect of NS3-K590R substitution on the domain 3 functionality of our study isolates is unclear. In summary, our analysis indicates that 2006 DENV-3 strains emerged from an ancestral strain that introduced into Southern Pakistan, possibly between 2001 and 2004. Pakistan DENV-3 strains evolved further to emerge as a distinct clade during the outbreak in Karachi in 2006.

### Characteristics of DENV-4 in Pakistan

**Figure 3:**
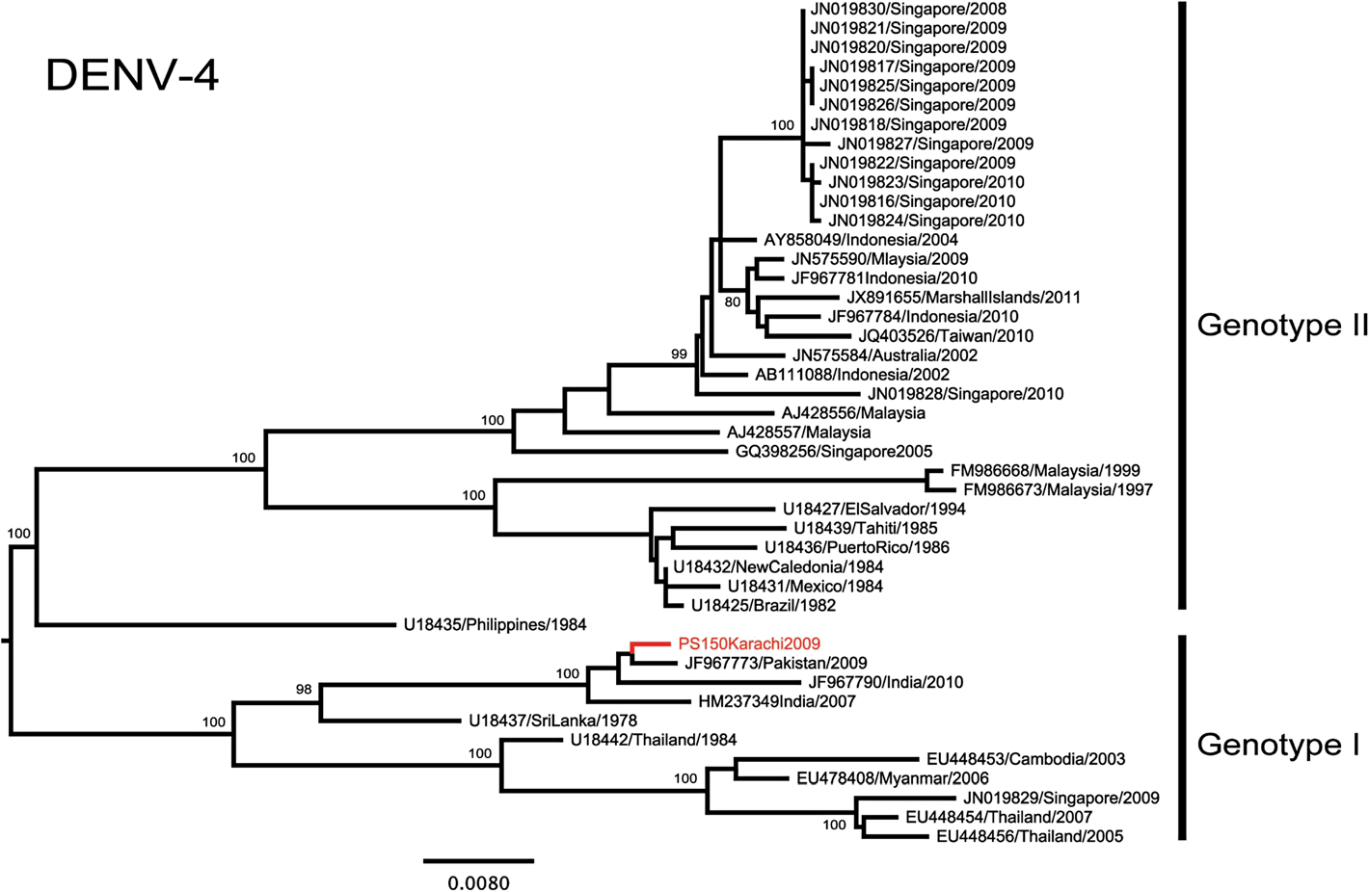
**Phylogenetic tree of DENV-4.** The maximum-likelihood tree was constructed based on the complete envelope gene sequences generated during this study and retrieved from GenBank. Sequences from Pakistan are highlighted in red. Figures on branches are bootstrap percentages. Only bootstrap values more than 75% are shown on the major nodes.

DENV-4 was first reported in Lahore during the 2008 dengue outbreak in Punjab, Pakistan [40]. Phylogenetic analysis showed that DENV-4 from the Indian subcontinent formed a distinct group within genotype I (Figure 3). A DENV-4 isolate obtained from Karachi in 2009 during our study clustered with another Pakistan sequence reported previously in the same year [41]. These Pakistan isolates shared the highest nucleotide (98.5%) and amino acid (99.3%) with a DENV-4 strain (GenBank: HM237349) from Andra Pradesh in Southren India in 2007 [42]. Therefore, it is evident that DENV-4 strains closely related to those in Pakistan in 2009 have previously been circulating in the Indian subcontinent. Nevertheless, more sequence data from a wider geographical scale in the Indian subcontinent region is needed to confirm the spread and evolutionary history of DENV-4 in Pakistan.

## Conclusions

DENV-2, DENV-3 and DENV-4 have co-circulated in Pakistan from 2008 to 2011. All three serotypes in Pakistan share an Indian subcontinent ancestry and are likely to have been introduced first into Southern Pakistan. DENV-2 and DENV-3 have undergone *in situ* evolution to emerge as distinct, heterogenous virus populations during the same period. While both DENV-2 and DENV-3 continued to circulate in Southern Pakistan until 2009, DENV-2 has subsequently spread in a Northern direction, a move that caused a massive outbreak in Punjab province in 2011. The study, therefore, highlights the implication of human migration and virus evolution in the expansion of dengue to areas where the disease was not previously a major public health threat, and the emergence of new strains that may be associated with severe outbreaks.

## Methods

### Sample collection

Aga Khan University Hospital (AKUH) is a 550 bed tertiary care hospital located in Karachi, Pakistan. The hospital is equipped with a state of the art clinical laboratory that caters not only for the in-patients but also reaches out to the community. It has a unique network of 200 sample collection units spread throughout the country. The laboratory receives samples from inpatients and outpatients of AKUH and from other clinics and hospitals in all major cities of the country. Karachi witnessed its first ever severe dengue outbreak in 2005–06 during which the clinical laboratory of AKUH was at the forefront of dengue diagnosis.

The patients presenting to the clinics and in-patients of the AKUH Karachi, with the history of fever, headache, joint pain, vomiting and diarrhoea were recruited based on presumptive clinical diagnosis of DENV infection. In addition, samples obtained from out patients at collection units were also included. After taking informed consent, a complete Performa about patient’s demographics and clinical presentation were filled. Serum samples of each patient were stored in three aliquots at −80°C. Two hundred dengue-suspected sera collected from 2006 to 2011 were retrieved from the serum bank at AKUH and were shipped to Environmental Health Institute, National Environment Agency, Singapore for the molecular epidemiological analysis. The work presented here was approved by the Ethical Review Committee, Aga Khan University (AKU), Pakistan.

### Virus isolation

DENV was isolated from sera using the *Ae. albopictus* clone C6/36 mosquito cell line (ATCC CRL-1660). Briefly, the monolayer of C6/36 was inoculated with serum in L-15 maintenance medium containing 3 % FCS at 33°C to allow for virus adsorption and replication. The infected fluid was harvested after 5–10 days. The presence of DENV in cell supernatants was confirmed by an immunofluoresent assay (IFA) during which cells were reacted with either DENV group-specific or serotype-specific monoclonal antibodies derived from hybridoma cultures (ATCC HB-46, HB-47, HB-48, and HB-49). The fluorescein isothiocyanate-conjugated goat anti-mouse antibody was used as the detector. A maximum of three passages was done for each sample based on the original virus load.

### RNA extraction and conventional PCR for detection and serotyping of Dengue virus

Viral RNA was extracted either from sera or culture supernatants using the QIAGEN QIAamp viral RNA minikit (QIAGEN, Hilden, Germany) according to the manufacturer’s recommendations.

Conventional semi-nested PCR was performed using a modified procedure described by Lanciotti and colleagues [43]. A one-step RT-PCR was performed using the AccessQuickTM RT-PCR System (Promega, Madison, WI, USA) in a 50 μl reaction volume containing 1X AccessQuick TM Master Mix, 2.5 units of AMV Reverse Transcriptase and 0.25 μM (each) of Primers D1 and D2 [43]. The reactions were carried out in Tgradient PCR Thermocycler (Biometra, Gottingen, Germany) using the following programme; reverse transcription at 45°C for 30 min, inactivation at 94°C for 2 min, and PCR amplification of 40 cycles under the following conditions: 94°C for 30 sec, 55°C for 30 sec, 72°C for 1 min, and a final extension at 72°C for 5 min. Five microlitres of the 1:100 dilution of the first-round PCR product was further amplified in a 50 μl reaction mixture containing 1X Green GoTaq® Flexi Buffer, 1.5 mM MgCl2, 0.2 mM dNTPs, 1.25 units of GoTaq® DNA Polymerase (Promega, Madison, WI, USA) and 0.5 μM of each primer D1, TS1, TS2, TS3 and TS4 [43]. After denaturation at 95°C for 2 min, the second round amplification was performed for 30 cycles (95°C for 30 sec, 55°C for 30 sec, 72°C for 1 min, and a final extension at 72°C for 10 min). Amplified products were visualized in 2% agarose gels stained with GelRed (Biotium Inc., USA).

### PCR amplification of envelope gene for sequencing

The synthesis of complementary DNA (cDNA) from RNA extracted from sera was carried out using Superscript™ III First-strand Synthesis System (Invitrogen, Carlsbad, USA). The complete envelope (E) gene (~1.5 kb) was amplified by PCR using 0.5 μM of DENV serotype-specific primers (Additional file 2) and 1X Phusion™ Flash High-Fidelity PCR Master Mix (Finnzymes, Lafayette, CO). The amplification protocol is as follows: initial denaturation at 98°C for 5 sec, 35 cycles of denaturation at 98°C for 5 sec, annealing at 60°C for 8 sec, extension at 72°C for 25 sec and final extension at 72°C for 1 min. Semi-nested PCR (~1.4 kb, Additional file 2) was performed as above if no desired product yielded in the first-round amplification. Amplified products were visualized in 2% agarose gels stained with GelRed (Biotium Inc., USA).

### Whole genome sequencing

A total of 13 DENV isolated from DENV-2 (n=6), DENV-3 (n=6) and DENV-4 (n=1) positive sera were selected for full genome sequencing. Isolates were selected to represent different groups of viruses based on E gene phylogeny. Amplification and sequencing primers were designed to cover the entire genome of different serotypes in overlapping fragments (Additional files 3, 4 and 5). Additional two sets of primers (Additional file 6) were used to capture 5' and 3' untranslated regions of complete genome for all four DENV serotypes. The PCR reaction was performed using the Phusion™ Flash High-Fidelity PCR Master Mix (Finnzymes, Lafayette, CO) as described for E gene amplification. Amplified products were visualized in 2% agarose gels stained with GelRed (Biotium Inc., USA).

### Purification and sequencing of PCR products

PCR products were purified using the Qiaquick PCR Purification kit (Qiagen, Hilden, Germany) according to manufacturer’s instructions. Sequencing of purified PCR products was perfromed by a commercial sequencing company (AITbiotech Pte Ltd, Singapore) according to the BigDye Terminator Cycle Sequencing kit (Applied Biosystems, USA) protocol. Seqeunces were deposited in Genbank database under the accession numbers KF041212 - KF041260.

### Phylogenetic and mutation analyses of envelope genes and complete genomes

Nucleotide sequences were assembled using the Lasergene package version 8.0 (DNASTAR Inc., Madison, WI, USA). Contiguous sequences were aligned using ClustalX program [44] and compared with published sequences of DENV isolates in Genbank database. Phylogenetic analysis of E gene sequences was performed in MEGA5 program [45] using the maximum-likelihood method based on the general time reversible (GTR) model with gamma distribution and invariant sites. The robustness of the original tree was tested with 1000 bootstrap replications. Comparison of amino acid sequences deduced from nucleotide alignments was performed by using the BioEdit v7.0.5 software to identify unique mutations in study isolates.

### Estimation of the time of viral emergence for DENV-2 and DENV-3

The time to the most recent common ancestor (TMRCA) was inferred using the Bayesian Markov Chain Monte Carlo (MCMC) method implemented in the BEAST package v1.7.4 [46,47]. A GTR substitution model with gamma distribution of rate variation among sites and a proportion of invariable sites, an uncorrelated log-normal relaxed molecular clock model and a Bayesian skyline coalescent model were used for this analysis. One hundred million generations of MCMC chains were run with sampling at every 10,000 generations. The MCMC output was analysed using the Tracer Version 1.5 programme (http://tree.bio.ed.ac.uk/software/tracer/). The uncertainty in parameter estimates was expressed as 95% highest probability density (HPD). All runs of MCMC were repeated to ensure convergence with effective sample size (ESS) of >200.

### Test for selection pressure

Genome-wide dN/dS ratios were computed in Hyphy 2.1.2 standalone version [29] using the single likelihood ancestor counting (SLAC), fixed effects likelihood (FEL), internal fixed effects likelihood (IFEL) and the mixed effect model of evolution (MEME) methods [30]. The Muse and Gaut (MG94) codon-based substitution model and GTR-based maximum likelihood phylogeny with gamma (4) distributed invariant sites, constructed using MEGA 5 [45], were used and significance levels were set at p<0.05.

## Abbreviations

DENV: Dengue virus; DHF: Dengue hemorrhagic fever; DSS: Dengue shock syndrome; E: Envelope; FEL: Fixed effects likelihood; GTR: General time reversible; HPD: Highest probability density; IFEL: Internal fixed effects likelihood; MCMC: Monte Carlo Markov Chain; MEME: mixed effect model of evolution; PCR: Polymerase chain reaction; SLAC: Single likelihood ancestor counting; TMRCA: Time to the most recent common ancestor.

## Competing interests

Authors declare that they have no competing interests.

## Authors’ contributions

EK and NLC conceived the study. EK actively recruited patient and analyzed patient demographic and clinical data, AN executed PCR, ZH contributed intellectual input in optimization of the PCR assays at AKU. CK performed virus isolation and generated sequence data. CK, HCH and KSL performed sequence analyses. EK, CK, HCH and KSL wrote the manuscript. All authors read and approved the final manuscript.

## Supplementary Material

Additional file 1: Table S1 Information of the samples sequenced. Click here for file

Additional file 2: Table S2 Serotype-specific primers used for amplification and sequencing of the envelope protein gene. Click here for file

Additional file 3: Table S3 Primers used for amplification and sequencing of complete genome of DENV-2. Click here for file

Additional file 4: Table S4 Primers used for amplification and sequencing of complete genome of DENV-3. Click here for file

Additional file 5: Table S5 Primers used for amplification and sequencing of complete genome of DENV-4. Click here for file

Additional file 6: Table S6 5’& 3’UTR primers used for amplification and sequencing of the complete genome of DENV. Click here for file
