# Supplementary material for: Evolution and heterogeneity of multiple serotypes of Dengue virus in Pakistan, 2006–2011
Source: Virol J. 2013 Sep 4;10:275. doi: 10.1186/1743-422X-10-275 (PMC3844417; doi:10.1186/1743-422X-10-275)
Supplement: Additional file 1: Table S1 — Information of the samples sequenced. [file 1743-422X-10-275-S1.doc]

**Table S1. Information on the samples sequenced.**

| **Serotype** | **Isolate ID** | **Genbank ID** | **Collection Year** | **Location** | **Clinical Classification** | **Age** | **Gender** | **Sequence**  **availability** |
| --- | --- | --- | --- | --- | --- | --- | --- | --- |
| DENV-2 | D2/Pakistan/2011-22/2011 | KF041223 | 2011 | Lahore | DF | 18 | M | E gene |
|  | D2/Pakistan/2011-23/2011 | KF041232 | 2011 | Lahore | DHF | 25 | M | Full genome |
|  | D2/Pakistan/2011-24/2011 | KF041224 | 2011 | Lahore | DHF | 65 | M | E gene |
|  | D2/Pakistan/2011-28/2011 | KF041225 | 2011 | Lahore | DHF | 59 | F | E gene |
|  | D2/Pakistan/2011-14/2011 | KF041219 | 2011 | Sahiwal | DF | 37 | F | E gene |
|  | D2/Pakistan/2011-17/2011 | KF041220 | 2011 | Lahore | DHF | 19 | F | E gene |
|  | D2/Pakistan/2011-19/2011 | KF041221 | 2011 | Lahore | DHF | 60 | F | E gene |
|  | D2/Pakistan/2011-20/2011 | KF041222 | 2011 | Lahore | DHF | 30 | M | E gene |
|  | D2/Pakistan/2011-3/2011 | KF041233 | 2011 | Multan | DHF | 29 | M | Full genome |
|  | D2/Pakistan/2011-4/2011 | KF041234 | 2011 | Lahore | DHF | 31 | F | Full genome |
|  | D2/Pakistan/2011-7/2011 | KF041226 | 2011 | Lahore | DHF | 40 | M | E gene |
|  | D2/Pakistan/171/2009 | KF041218 | 2009 | Karachi | DHF | 58 | F | E gene |
|  | D2/Pakistan/209/2009 | KF041235 | 2009 | Karachi | DF | 24 | M | Full genome |
|  | D2/Pakistan/154/2009 | KF041217 | 2009 | Karachi | DHF | 34 | F | E gene |
|  | D2/Pakistan/132/2009 | KF041214 | 2009 | Karachi | DF | 28 | M | E gene |
|  | D2/Pakistan/140/2009 | KF041215 | 2009 | Sachal Goth | DSS | 40 | M | E gene |
|  | D2/Pakistan/148/2009 | KF041216 | 2009 | Karachi | DHF | 23 | M | E gene |
|  | D2/Pakistan/125/2009 | KF041212 | 2009 | Karachi | DSS | 27 | M | E gene |
|  | D2/Pakistan/130/2009 | KF041213 | 2009 | Karachi | DHF | 26 | M | E gene |
|  | D2/Pakistan/78/2009 | KF041237 | 2009 | Karachi | DSS | 23 | F | Full genome |
|  | D2/Pakistan/79/2009 | KF041231 | 2009 | Jacobabad | DHF | 22 | M | E gene |
|  | D2/Pakistan/82/2009 | KF041230 | 2009 | Karachi | DF | 42 | M | E gene |
|  | D2/Pakistan/51/2009 | KF041236 | 2009 | Karachi | DF | 21 | M | Full genome |
|  | D2/Pakistan/55/2008 | KF041228 | 2008 | Karachi | DHF | 23 | M | E gene |
|  | D2/Pakistan/64718/2009 | KF041229 | 2009 | Karachi | DF | 30 | M | E gene |
|  | D2/Pakistan/232/2009 | KF041227 | 2009 | Karachi | DHF | 35 | M | E gene |
| DENV-3 | D3/Pakistan/55709/2006 | KF041256 | 2006 | Karachi | DF | 25 | M | Full genome |
|  | D3/Pakistan/56609/2006 | KF041239 | 2006 | Karachi | DF | 31 | M | E gene |
|  | D3/Pakistan/55445/2006 | KF041240 | 2006 | Karachi | DF | 20 | F | E gene |
|  | D3/Pakistan/55857/2006 | KF041253 | 2006 | Karachi | DF | 4 | M | E gene |
|  | D3/Pakistan/55400/2007 | KF041241 | 2007 | Karachi | DF | 21 | F | E gene |
|  | D3/Pakistan/55505/2007 | KF041255 | 2007 | Hyderabad | DF | 25 | F | Full genome |
|  | D3/Pakistan/54241/2006 | KF041242 | 2006 | Karachi | DF | 60 | F | E gene |
|  | D3/Pakistan/54155/2006 | KF041243 | 2006 | Karachi | DHF | 24 | F | E gene |
|  | D3/Pakistan/52440/2006 | KF041257 | 2006 | Karachi | DF | 23 | M | Full genome |
|  | D3/Pakistan/51741/2006 | KF041245 | 2006 | Karachi | DHF | 42 | F | E gene |
|  | D3/Pakistan/56139/2006 | KF041238 | 2006 | Karachi | DF | 30 | M | E gene |
|  | D3/Pakistan/46849/2006 | KF041244 | 2006 | Karachi | DF | 40 | F | E gene |
|  | D3/Pakistan/44454/2006 | KF041246 | 2006 | Karachi | DF | 30 | M | E gene |
|  | D3/Pakistan/43298/2006 | KF041259 | 2006 | Karachi | DHF | 10 | F | Full genome |
|  | D3/Pakistan/40044/2006 | KF041247 | 2006 | Karachi | DF | 34 | M | E gene |
|  | D3/Pakistan/36025/2006 | KF041249 | 2006 | Karachi | DF | 43 | M | E gene |
|  | D3/Pakistan/33694/2006 | KF041248 | 2006 | Karachi | DHF | 35 | M | E gene |
|  | D3/Pakistan/33004/2006 | KF041250 | 2006 | Karachi | DF | 21 | M | E gene |
|  | D3/Pakistan/173/2009 | KF041252 | 2009 | Karachi | DF | 21 | F | E gene |
|  | D3/Pakistan/71/2009 | KF041251 | 2009 | Karachi | DF | 12 | M | E gene |
|  | D3/Pakistan/56/2008 | KF041254 | 2008 | Karachi | DF | 44 | M | Full genome |
|  | D3/Pakistan/45251/2009 | KF041258 | 2009 | Karachi | DF | 19 | F | Full genome |
| DENV-4 | D4/Pakistan/150/2009 | KF041260 | 2009 | Karachi | DF | 21 | F | Full genome |

DENV=Dengue Virus, DF=Dengue fever, DHF=Dengue haemorrhagic fever, E gene=Envelope gene, NA=Not available

All DENV-2 isolates belong to Cosmopolitan genotype (Indian lineage). All DENV-3 isolates are of genotype III. The DENV-4 isolate belongs to Genotype I.
