# Supplementary material for: Evolution and heterogeneity of multiple serotypes of Dengue virus in Pakistan, 2006–2011
Source: Virol J. 2013 Sep 4;10:275. doi: 10.1186/1743-422X-10-275 (PMC3844417; doi:10.1186/1743-422X-10-275)
Supplement: Additional file 2: Table S2 — Serotype-specific primers used for amplification and sequencing of the envelope protein gene. [file 1743-422X-10-275-S2.doc]

**Table S2.** Serotype-specific primers used for amplification and sequencing of the envelope protein gene.

| **Serotype** | **Primer** | **Sequence (5’ – 3’)** |
| --- | --- | --- |
| DENV-1 | D1-816F*^§^ | CTCTGAGACACCCAGGATTCAC |
|  | D1-2598R* | GCTGATCGAATTCCACACAC |
|  | D1-2474R^§^ | CCRCTTCCACATTTRAGYTCTC |
|  | D1-1229F | AGAGGCTGGGGCAATGG |
|  | D1-1710R | GCTCCTTCTTGTGATCCTAGTAC |
| DENV-2 | D2-851F*^§^ | CAATCCTGGCATACACCATAG |
|  | D2-2540R* | GAAGGGGATTCTGGTTGG |
|  | D2-2364R^§^ | AGACAGTGAKGTGCTRCGTGA |
|  | D2-1353F | GTGATAACACCTCACTCAGGG |
|  | D2-1298R | CCTATAGATGTGAACACTCCTCC |
| DENV-3 | D3-793F*^§^ | AGTCGAGAAGTAGAGACATGG |
|  | D3-2503R* | CTCTGTCCAGGTGTGGACCT |
|  | D3-2365R^§^ | GCATGAAAATGACATRGAWGTGT |
|  | D3-1307F | ATAGAGGGAAAAGTGGTGCA |
|  | D3-1867R | GGAGACTTCTTTCTTCAACACAAA |
| DENV-4 | D4-896F* | CTGTTTTCTTTGTCCTAATGATGCT |
|  | D4-922F^§^ | TCGCYCCATCCTACGGAATGC |
|  | D4-2434R*^§^ | AACCCATGTCTGCTTGAACTGTGA |
|  | D4-1954R | TCTCTATGGGGARTTTACACG |
|  | D4-1760F | ATGGAAATCACATGTTTGCAGGACA |

*First-round amplification primers; ^§^Semi-nested amplification primers.
