# Supplementary material for: Evolution and heterogeneity of multiple serotypes of Dengue virus in Pakistan, 2006–2011
Source: Virol J. 2013 Sep 4;10:275. doi: 10.1186/1743-422X-10-275 (PMC3844417; doi:10.1186/1743-422X-10-275)
Supplement: Additional file 3: Table S3 — Primers used for amplification and sequencing of complete genome of DENV-2. [file 1743-422X-10-275-S3.doc]

**Table S3.** Primers used for amplification and sequencing of complete genome of DENV-2.

| **Primer Name** | **Sequence (5' - 3')** |
| --- | --- |
| Den2-16F* | TGGACCGACAAAGACAGATTCTTTG |
| Den2-348F | CAGGAAAGAGATTGGAAGGATGC |
| Den2-647R | TGCACCAACARTCTATGTCTTCTG |
| Den2-1169R* | CCTTGTGTTGGGCAGCGAGAT |
| Den2-851F* | CAATCCTGGCATACACCATAG |
| Den2-1353F | GTGATAACACCTCACTCAGGG |
| Den2-1596R | CCATGGTAACGGCAGGTC |
| Den2-1746F | CCAGATGTCGTCAGGAAACTTAC |
| Den2 2540R* | GAAGGGGATTCTGGTTGG |
| Den2-2146F* | ACAACAATGAGAGGAGCGAAGAG |
| Den2-2796F | CACAGAACTTCATAACCACACCT |
| Den2-3054R | TCTTCCATGTGTCATTGAGTGC |
| Den2-3480R | TCCGGCTGTGACCAAAGAGTT |
| Den2-3865R | CTAAAGCCAACGCGTCAGTCAGT |
| Den2-3545F | TCAGGACCCGAGTAGGAACGAA |
| Den2-4158F* | GGCAGTCGGGATGGTGAGC |
| Den2-4746F | CTAATATCRTATGGAGGAGG |
| Den2-4590R* | ATAGGCTCCATCTTCCAGTTCAG |
| Den2-5403R | TATGCTTGCTGGGTCTGTGAAAT |
| Den2-5784R* | CCTCTCGGCCTTGAAGTTAGCAC |
| Den2-5443F* | GAAGCAGCTGGGATTTTTATGAC |
| Den2-6072R | GTCCACCTTTTCACGCTCTG |
| Den2-6161F | CTACARAGTGGCRGCTGAAGG |
| Den2-6776R | AATTGGTTATCTTGGGGTGTTC |
| Den2-6813F | GCAACCATGGCAAACGAGATG |
| Den2-7392R | AGCCCATGTAGTCCTCATCAT |
| Den2-7733R* | ACAGCGTGATGGTCCGTTTCT |
| Den2-7416F* | AACCGGGCCCATCTCTACACTG |
| Den2-7926F | CGGTTGGAATCTGGTGCGTCTT |
| Den2-8181R | AGCTCCTCCGTATTTCCTTTGT |
| Den2-8327F | AGCCAGATGTTGACCTCGGAAGT |
| Den2-8847R | AACAGCCTCACGTGCCGACTT |
| Den2-8662F* | GAACCGAAAGAAGGCACGAAAAA |
| Den2-9232F | ACCACATGARAGGAGAACACAA |
| Den2-9154R* | CTCCTCCTTCTTTCTTACTCACG |
| Den2-9599R | GCGCTTGCAAACCTGTCATCTAA |
| Den2-9821F | CAGCCTGTTTGGGGAAGTCT |
| Den2-10321R | GGCTTAATCCGACCTGACTTCTG |
| Den2-10695R * | CATTTTCTGGCGTTCTGTGC |

Amplification primers are denoted by asterisks. The remaining primers, together with amplification primers were used for sequencing.
