# Supplementary material for: Evolution and heterogeneity of multiple serotypes of Dengue virus in Pakistan, 2006–2011
Source: Virol J. 2013 Sep 4;10:275. doi: 10.1186/1743-422X-10-275 (PMC3844417; doi:10.1186/1743-422X-10-275)
Supplement: Additional file 4: Table S4 — Primers used for amplification and sequencing of complete genome of DENV-3. [file 1743-422X-10-275-S4.doc]

**Table S4.** Primers used for amplification and sequencing of complete genome of DENV-3.

| **Primer Name** | **Sequence (5' - 3')** |
| --- | --- |
| Den3-80F* | GAGAGCAGATCTCTGATGAACAAC |
| Den3-470R | TGCGCGGCTCTCCATCTCGT |
| Den3-611F | GTGGARCCTGAAGACATTGACTGC |
| Den3-1093R | CAGTTGGGTGGCCTCGGTCTT |
| Den3-1594R* | TGATGYCCATGGTARAGGTAGGTC |
| Den3-1084F* | CACCCAACTGGCGACCCTAAG |
| Den3-1585F* | ATGGRCATCAGGRGCTACAACAGA |
| Den3-1768R | CGCRAARATRCTTGTGCCTCCTG |
| Den3-2448R* | TCTTTGCCYTTCCAGTTTATGACA |
| Den3-2111F | GGRAGCTCGATTGGGAAGATGTTT |
| Den3-2515F | YCAAGCAGATTCCCCHAAAAGAYT |
| Den3-2770R | TCCCCAYGTTTTCCATGARTATTT |
| Den3-3187R* | GGGCCTGTARRTGTGTTGYGARAT |
| Den3-3075F* | AAACCTGCACATGGCCAAAATCAC |
| Den3-3866R | CCATRAGCCCCARRGCDATTCC |
| Den3-3987F | CCGGARTTTCGCTTTTRCCAGTGT |
| Den3-4478R* | AVARTGTKGCRGGTATGGAGTATG |
| Den3-4120F* | GAGAAGCTGGCCACTGAATGARGG |
| Den3-4782R | TGCCATTGTGCRCTCARTCTCC |
| Den3-5065F | CAAAAAGCGAAAYCTRACCATAA |
| Den3-5399R | CTATRCTGGCTGGRTCTGTGAAAT |
| Den3-6103R* | GGACTCACCYTTCAGGCGRTAYTC |
| Den3-5613F* | AAGCYGGRAATGACATAGCAAACT |
| Den3-6081F | ARTAYCGCCTGAARGGTGAGTCCA |
| Den3-6356F | GCRGCTGGYAGAAAGTCRATC |
| Den3-6491R | ARGCCCTMCCRCCRTGTTCTGACG |
| Den3-6888R* | GAAACAACACCTGGYTCYTTRGAC |
| Den3-6763F* | CAAYCAACTCGCATATGTCGTGAT |
| Den3-7353R | GCRCACARRACCAGGAGCATAAC |
| Den3-7530F | TTTCTATYATGAAATCAGTTGGAA |
| Den3-7816F | AGGAGGCTGGTCATATTAYTGTGC |
| Den3-8460R | TARGGRTTTTCRTCATCATAGTGC |
| Den3-8790R* | CCCATRGCTGCGTTRGTTCTGACC |
| Den3-8573F* | GTGACACAGATGGCAATGACRGAY |
| Den3-9319R* | CYTTGGAGTYGGTCGTTGAA |
| Den3 8963F* | GCTATATGGTACATGTGGTTGGG |
| Den3 8998F | GGGAGCCAGGTACCTTGAGTTCG |
| Den3 9794F | GCRGGATGGAGCCTTARAGAAACT |
| Den3 9841R | RGCGTAGGCTTTCCCYAGRCAT |
| Den3 10356R | TCCTTGGACGGGGCTCACAG |
| Den3 10497R* | CTGCTGCGTTGTGTCATGG |

Amplification primers are denoted by asterisks. The remaining primers, together with amplification primers were used for sequencing.
