# Supplementary material for: Evolution and heterogeneity of multiple serotypes of Dengue virus in Pakistan, 2006–2011
Source: Virol J. 2013 Sep 4;10:275. doi: 10.1186/1743-422X-10-275 (PMC3844417; doi:10.1186/1743-422X-10-275)
Supplement: Additional file 5: Table S5 — Primers used for amplification and sequencing of complete genome of DENV-4. [file 1743-422X-10-275-S5.doc]

**Table S5.** Primers used for amplification and sequencing of complete genome of DENV-4.

| **Primer Name** | **Sequence (5' - 3')** |
| --- | --- |
| Den4-40F* | TCGGAAGCTTGCTTAACACAGTTCT |
| Den4-514F | TCYAGACAACAGAARGARTCAAC |
| Den4-644R | GCACCAGCAATCAATGTCTTCAGG |
| Den4-895F | CTWTCYTCTTTGTCYTAATGATGCT |
| Den4-1049R | TCCCTGGGCCATGGTTGTGAYG |
| Den4-1449R | CCGATGGTGACCTGGYAGTTA |
| Den4-1462F | YARCGGACTATGGAGAAYTAACAC |
| Den4-1601F | AGCAGGAGCAGACYCATCAGAARTT |
| Den4-1939R | TTACACGGAGCTCCAGYACCTTCAT |
| Den4-2340R* | TGCCAATCCACARCACTAAGAA |
| Den4-2053F* | CCYTTGGGGACAGTTACYTAG |
| Den4-2513F | ACAGTACAAATTTYAACCAGAGTCC |
| Den4-2887F | TGRAAGACTATGGATTTGGTYTGTT |
| Den4-3312R | ATGGGCCTCTATGGTCACAATCCTC |
| Den4-3616F | GAGGCYTCACATGGATGGACTTART |
| Den4-3812R | CGTTGTCATGGCCATTCCTATTACC |
| Den4-4084F* | AARCTCYGCCAGTGTACCTAATGAC |
| Den4-4477R* | GGAATTGCCAAGGYATYGAGACC |
| Den4-4888R | TCTCCAGTTAGGGRCTTGAAA |
| Den4-5542F | CARAAAGGTCATGGAATYCAGG |
| Den4-5933R | GTYGTCTTCTTGTGCTGYGTTCCWA |
| Den4-6000F | ATGCTGCTTGACYATATCTACACC |
| Den4-6524R* | CGYGAGTTCGTTCAGGGCGTGTT |
| Den4-6378F* | AGCYTAACTYTTYACATCCTAACA |
| Den4-6750F | AAACAAAGGACCCCACAAGACAACY |
| Den4-6971F | GCTGAGACATYCCATAGAAAACACG |
| Den4-7003R | TMGGKCGACGTGTTTTCTATGGYAT |
| Den4-7253F | CACGVTGGACGGGATAACAGTADTA |
| Den4-7515F | GCTGGACTGRCTYTTTCARTCATA |
| Den4-7560R | TCCYAGGGGTTTGTGCATTCTTTAT |
| Den4-8238R* | CCGAYGHACCTGACACCCYATACAT |
| Den4-7945F* | CAGGGGTTGACYTGTTCTAC |
| Den4-8335F | TTGGGRCAGGAACGAGAAGTGTC |
| Den4-8663R | TTTTGGTTVGGGTGTTCTGGYATDT |
| Den4-8752F | AAGAGTTCATTYCAAAAGTTAGGRC |
| Den4-9147R | YGTCTCCATCCTTCTTGTCTA |
| Den4-9167R | AGCAGGCTGGGACACARGAAT |
| Den4-9679F | AGGTTCCTTTTTGCTCTYATYACYT |
| Den4-9679R | TCTTGCCAGTTTTTCCATCCCTTYG |
| Den4-10116R | TCYGGGATCCACACCACAAYTC |
| Den4-10160F | GRCCATAACCYAGGTCAGGAACYYG |
| Den4-10392F | CGGAAGCTGTACGCGTGGTTTATTG |
| Den4-10612R* | GCGCTCTRTGCCTGGATTGAT |

Amplification primers are denoted by asterisks. The remaining primers, together with amplification primers were used for sequencing.
