# Supplementary material for: Evolution and heterogeneity of multiple serotypes of Dengue virus in Pakistan, 2006–2011
Source: Virol J. 2013 Sep 4;10:275. doi: 10.1186/1743-422X-10-275 (PMC3844417; doi:10.1186/1743-422X-10-275)
Supplement: Additional file 6: Table S6 — 5’& 3’UTR primers used for amplification and sequencing of the complete genome of DENV. [file 1743-422X-10-275-S6.doc]

**Table S6.** 5’& 3’UTR primers used for amplification and sequencing of the complete genome of DENV

| **Region** | **Primer** | **Sequence (5’ – 3’)** |
| --- | --- | --- |
| 5’ UTR | DENV_5UTRF* | GTAAAACGACGGCCAGTTGTTAGTCTRYGTGGACC |
|  | DENV_5UTRR*^§^ | GCGTTTCAGCATATTGAWAG |
|  | Universal seq primer M13F(-20) ^§^ | GTAAAACGACGGCCAGT |
| 3’ UTR | DENV123_3UTRF*^§^ | ATGMCWTCMATGAARAGATT |
|  | DENV4_3UTRF*^§^ | GCCAGTMATGAAAAGATACA |
|  | DENV_3UTRR* | GTAAAACGACGGCCAGTAGAACCTGTTGATTCAAC |
|  | Universal seq primer M13F(-20)^§^ | GTAAAACGACGGCCAGT |

*Amplification primers; ^§^Sequencing primers.
